# The effect of citrulline and arginine supplementation on lactic acidemia in MELAS syndrome^☆^

**DOI:** 10.1016/j.mgene.2013.09.001

**Published:** 2013-10-15

**Authors:** Ayman W. El-Hattab, Lisa T. Emrick, Kaitlin C. Williamson, William J. Craigen, Fernando Scaglia

**Affiliations:** a Division of Medical Genetics, Department of Pediatrics, The Children's Hospital, King Fahad Medical City, Riyadh, Saudi Arabia; b Faculty of Medicine, King Saud bin Abdulaziz University for Health Sciences, Riyadh, Saudi Arabia; c Department of Molecular and Human Genetics, Baylor College of Medicine, Houston, TX, USA

**Keywords:** Mitochondrial myopathy, Stroke-like episodes, Lactic acidosis, Endothelial dysfunction, Nitric oxide (NO), m.3243A>G heteroplasmy

## Abstract

Mitochondrial encephalomyopathy, lactic acidosis, and stroke-like episodes (MELAS) syndrome is a mitochondrial disorder in which nitric oxide (NO) deficiency may play a role in the pathogenesis of several complications including stroke-like episodes and lactic acidosis. Supplementing the NO precursors arginine and citrulline restores NO production in MELAS syndrome. In this study we evaluated the effect of arginine or citrulline on lactic acidemia in adults with MELAS syndrome. Plasma lactate decreased significantly after citrulline supplementation, whereas the effect of arginine supplementation did not reach statistical significance. These results support the potential therapeutic utility of arginine and citrulline in MELAS syndrome and suggest that citrulline supplementation may be more efficacious. However, therapeutic efficacy of these compounds should be further evaluated in clinical trials.

## Highlights

• MELAS syndrome is a common mitochondrial disease.
• Nitric oxide (NO) deficiency in MELAS may aggravate lactic acidosis.
• Arginine and citrulline are NO precursors and can restore NO production in MELAS.
• We assessed the effect of arginine and citrulline on lactate in adults with MELAS.
• Lactate decreased with citrulline or arginine with more reduction after citrulline.

## 1 Introduction

Mitochondrial encephalomyopathy, lactic acidosis, and stroke-like episodes (MELAS) syndrome is one of the most frequent maternally inherited mitochondrial disorders (Chinnery and Turnbull, 2011). It is caused by mutations in mitochondrial DNA, with the most common mutation being the m.3243A>G mutation in the *MTTL1* gene that encodes tRNA^Leu/(UUR)^ (Wallace, 1992). MELAS syndrome has a broad spectrum of manifestations, including stroke-like episodes, exercise intolerance, muscle weakness, epilepsy, dementia, migraine headaches, short stature, sensorineural hearing loss, lactic acidosis, and diabetes (Hirano and Pavlakis, 1994). No consensus recommendations have been established for its treatment, although arginine supplementation is commonly used based upon uncontrolled open label clinical studies. The only published randomized placebo controlled clinical trial for MELAS syndrome used dichloroacetate and had to be terminated early due to toxicity (Kaufmann et al., 2006). In addition, several medications including antioxidants, respiratory chain substrates, and cofactors in the form of vitamins are used with no proven efficacy (Scaglia and Northrop, 2006).

Energy depletion due to mitochondrial dysfunction can explain many of the multi-organ manifestations of MELAS syndrome including myopathy, epilepsy, and diabetes (Wallace, 1999). In addition to reduced energy production, there has been growing evidence that nitric oxide (NO) deficiency occurs in MELAS syndrome and can play a major role in the pathogenesis of several complications including stroke-like episodes, myopathy, diabetes, and lactic acidosis (Naini et al., 2005, Koga et al., 2005, Koga et al., 2006, Koga et al., 2007, Tengan et al., 2007, Sproule and Kaufmann, 2008, Vattemi et al., 2011, El-Hattab et al., 2012a, El-Hattab et al., 2012b). The amino acids arginine and citrulline act as NO precursors and their administration has been shown to restore NO production in MELAS syndrome, with citrulline having a greater effect (El-Hattab et al., 2012a). Therefore, arginine and citrulline can potentially be of therapeutic utility in treating NO deficiency-related manifestations in this syndrome. Arginine supplementation to individuals with MELAS syndrome is reported to result in an improvement in clinical symptoms associated with stroke-like episodes and a decrease in the frequency and severity of these episodes (Koga et al., 2005, Koga et al., 2006, Koga et al., 2007). However, there are no clinical studies evaluating the effect of arginine or citrulline supplementation on other MELAS manifestations where NO deficiency may play a role.

Lactic acidemia occurs in more than 90% of subjects with MELAS syndrome (Hirano and Pavlakis, 1994). Lactic acidosis results from an inability of dysfunctional mitochondria to generate sufficient ATP, leading to shunting of pyruvate to lactate (Sproule and Kaufmann, 2008). Moreover, hypoperfusion may result in lactic acidosis due to decreased oxygen delivery to peripheral tissues and a shift to anaerobic glycolysis. NO deficiency in MELAS syndrome can result in decreased blood perfusion and therefore may aggravate lactic acidosis. In this study we hypothesized that arginine and citrulline supplementation would result in lowering plasma lactate levels in subjects with MELAS syndrome via increasing NO availability and improving perfusion. Herein, we compared plasma lactate concentrations before and after short-term oral arginine or citrulline supplementation to adults with MELAS syndrome.

## 2 Material and methods

We evaluated 10 adults aged 18–57 years, diagnosed clinically with MELAS syndrome, and harboring the m.3243A>G mutation. Blood samples for plasma lactate measurement were withdrawn from each subject before and after 48 h supplementation of oral l-arginine at a dose of 10 g/m^2^ body surface area/day divided every 4 h. After an interval of at least 1 week, the study was repeated before and after l-citrulline supplementation with the same dose, frequency, and duration. In addition, we studied 10 healthy control subjects aged 20–46 years for whom blood samples were withdrawn once for plasma lactate measurement. For each subject with MELAS syndrome an additional blood sample and a urine sample were obtained to assess the heteroplasmy of the m.3243A>G mutation. These cohorts are the same cohorts enrolled in our previous study to assess NO production (El-Hattab et al., 2012a).

The blood samples were drawn into heparinized tubes and centrifuged immediately. The plasma was transferred and stored at − 70 °C for later analyses. Plasma lactate concentrations were measured with the YSI 2300 Stat Plus analyzer (YSI Life Sciences, Yellow Springs, OH, USA) according to the manufacturer's instructions. Heteroplasmy of the m.3243A>G mutation in blood and urine was measured using the amplification refractory mutation systems quantitative PCR (ARMS-qPCR) method, which provides simultaneous detection and quantification of heteroplasmic mitochondrial DNA point mutations (Bai and Wong, 2004).

Results were expressed as means ± SEMs. The results of the two baseline plasma lactate concentrations (before arginine and citrulline supplementation) for subjects with MELAS syndrome were averaged and compared to the values of the control subjects using the unpaired Student's *t* test. The differences between the plasma lactate concentrations before and after arginine or citrulline supplementation in subjects with MELAS syndrome were assessed by the paired Student's *t* test. Correlations between different value sets were assessed using Pearson Product-Moment Correlation Coefficient. Results were considered statistically significant if *p* < 0.05.

## 3 Results

The baseline plasma lactate for subjects with MELAS syndrome before arginine supplementation ranged from 1.67 to 4.72 mmol/L with average being 3.16 ± 0.29 mmol/L, and before citrulline supplementation ranged from 1.39 to 4.57 mmol/L with average being 3.17 ± 0.32 mmol/L. To evaluate the fluctuations of plasma lactate levels at baseline, we calculated the correlation between the plasma lactate values before arginine and citrulline supplementation and found a correlation coefficient of 0.66, which is statistically significant (*p* < 0.05) indicating minimal fluctuations. Only one subject with MELAS syndrome had baseline plasma lactate levels within the normal range (normal = 0.2–2.0 mmol/L); however, the remaining nine subjects had elevated baseline plasma lactate values before supplementation with either arginine or citrulline.

Plasma lactate for the healthy control subjects ranged from 0.70 to 1.30 mmol/L, with the average being 0.93 ± 0.06 mmol/L. The mean baseline plasma lactate (before arginine and citrulline supplementation) for subjects with MELAS was 3.17 ± 0.28 mmol/L. That value is more than three-fold higher than the value of control subjects and reaches strong statistical significance (*p* < 0.0005).

Following 48 h of supplementation, in subjects with MELAS syndrome the average plasma lactate concentration was lower both after arginine supplementation (3.16 → 2.99 mmol/L) and citrulline supplementation (3.17 → 2.94 mmol/L). However, this reduction was statistically significant after citrulline (*p* < 0.05), but not after arginine supplementation (Table 1).

**Table 1:** Plasma lactate concentration for 10 adults with MELAS syndrome before and after arginine and citrulline supplementation.

	Mean ± SEM	P value	Reduction %
Lactate before arginine (mmol/L)	3.16 ± 0.29	NS	5.4%
Lactate after arginine (mmol/L)	2.99 ± 0.28		
Lactate before citrulline (mmol/L)	3.17 ± 0.32	< 0.05	7.3%
Lactate after citrulline (mmol/L)	2.94 ± 0.33		

NS: not statistically significant, SEM: standard error of the mean.

In subjects with MELAS syndrome, the m.3243A>G mutation heteroplasmy ranged from 1 to 82% in blood and from 3 to 99% in urine. The correlation between the plasma lactate concentration and the m.3243A>G mutation heteroplasmy showed a statistically significant positive correlation, with a correlation coefficient of 0.59 (*p* < 0.05) between plasma lactate level and heteroplasmy in blood, and a correlation coefficient of 0.73 (*p* < 0.01) between plasma lactate level and heteroplasmy in urine (Table 2).

**Table 2:** Correlations between mean baseline plasma lactate concentrations and m.3243A>G heteroplasmy in blood and urine in subjects with MELAS syndrome.

Average baseline plasma lactate (mmol/L)	m.3243A>G heteroplasmy (%)	
	Blood	Urine
3.4	61	98
3.8	35	96
3.8	30	81
2.2	53	86
3.6	82	99
3.0	58	96
4.4	71	98
1.5	1	3
3.5	81	90
2.4	30	76
Correlation coefficient with plasma lactate	0.59 (*p* < 0.05)	0.73 (*p* < 0.01)

## 4 Discussion

Lactic acidemia is one of the cardinal signs of MELAS syndrome (Hirano and Pavlakis, 1994). Nine out of ten subjects with MELAS syndrome in this study had elevated plasma lactic acid, with the average baseline lactate being three fold higher than the values observed in healthy control subjects.

Although energy depletion due to mitochondria dysfunction in MELAS syndrome can explain many of its multi-organ manifestations, NO deficiency can play a major role in the pathogenesis of several complications including stroke-like episodes, myopathy, and lactic acidosis (Naini et al., 2005, Koga et al., 2005, Koga et al., 2006, Koga et al., 2007, Tengan et al., 2007, Sproule and Kaufmann, 2008, Vattemi et al., 2011, El-Hattab et al., 2012a, El-Hattab et al., 2012b). NO is formed from arginine via the enzyme nitric oxide synthase, which catalyzes the conversion of arginine to citrulline. Citrulline can be converted to arginine via argininosuccinate synthase and argininosuccinate lyase. Therefore, both arginine and citrulline act as NO precursors in a wide variety of cells including vascular endothelium (Förstermann et al., 1994, Villanueva and Giulivi, 2010, Mori and Gotoh, 2000). NO deficiency in MELAS syndrome may be due to multiple factors. These include impaired NO production due to a generalized impairment of endothelial function associated with mitochondrial proliferation in vascular endothelial cells (Ohama et al., 1987, Goto et al., 1992, Toda and Okamura, 2003, Green et al., 2004). Mitochondrial proliferation may also lead to NO sequestration due to increased cytochrome c oxidase (COX). In addition, there may be shunting of NO into reactive nitrogen species formation (Naini et al., 2005, Sproule and Kaufmann, 2008, Vattemi et al., 2011). Finally, there may be decreased availability of the NO precursors arginine and citrulline (Naini et al., 2005, Koga et al., 2006, El-Hattab et al., 2012a).

**Fig. 1:**
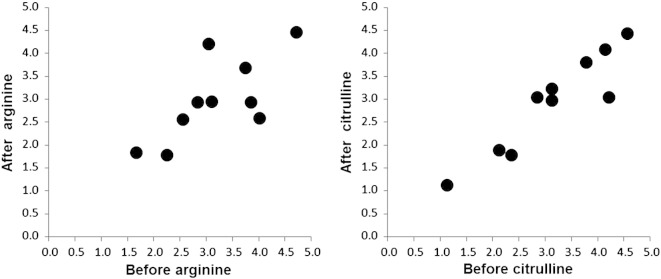
Scatter plot for the individual lactate values before and after arginine and before and after citrulline supplementations. Each point represents each research subject with plasma lactate level before supplementation plotted against the X axis and after supplementation plotted against the Y axis.

The results of this study demonstrate a statistically significant reduction in plasma lactate after citrulline supplementation. Furthermore, by plotting the individual plasma lactate values before and after arginine and citrulline supplementation using scatter plots, a reduction in the lactate levels is more consistent after citrulline supplementation; whereas, lactate reduction after arginine supplementation is more variable (Fig. 1). We have previously demonstrated that both arginine and citrulline supplementation increase the NO production rate in patients with MELAS syndrome, with citrulline resulting in a greater increase (El-Hattab et al., 2012a). In this study, the more significant and consistent lactate reduction observed after citrulline supplementation could be explained by the superior efficacy of citrulline in increasing NO production, leading to a better perfusion and lower lactate levels. These results also suggest that citrulline may have a better therapeutic effect.

The observed reduction in lactate after citrulline or arginine supplementation is relatively small and the degree of lactate reduction per se is unlikely to be of any clinical significance. However, the treatment in this study was limited to 48 h, and it is possible that longer administration of arginine or citrulline may have a greater impact on lactate levels. These results, along with the studies demonstrating beneficial effect of arginine supplementation on stroke-like episodes (Koga et al., 2005, Koga et al., 2006, Koga et al., 2007), provide more evidence for the potential therapeutic utility of arginine and citrulline in reversing the NO deficiency in MELAS syndrome. Increasing NO availability will potentially improve perfusion in all microvasculature compartments. Therefore, the effect of arginine and citrulline supplementation may not be limited to improving stroke-like episodes and lactic acidemia, but may also lead to improvement in other manifestations in which NO deficiency plays a role, including migraine headaches, muscle weakness, exercise intolerance, and diabetes. Additional assessments of the clinical effects of long-term arginine or citrulline supplementation on different aspects of MELAS syndrome are needed.

In our previous study we also demonstrated that subjects with MELAS syndrome have hyperalaninemia that improved after arginine (532 ± 23 → 496 ± 26 *p* < 0.05) and citrulline (536 ± 19 → 434 ± 28 *p* < 0.001) supplementations, with the reduction in alanine levels being more statistically significant after citrulline supplementation (El-Hattab et al., 2012a). The hyperalaninemia in subjects with MELAS syndrome is believed to be a consequence of lactic acidemia, and the reduction in the alanine level suggested improvement in lactic acidemia (El-Hattab et al., 2012a). In this study we present direct evidence that lactate is lower after citrulline and arginine supplementation. However, by comparing the effect of citrulline and arginine on alanine versus lactate levels we note that the reduction in alanine is more pronounced than that of lactate after citrulline (19% reduction in alanine and 7% reduction in lactate) and arginine (7% reduction in alanine and 5% reduction in lactate). In addition, after arginine supplementation, the reduction in alanine is statistically significant; however, the reduction in lactate is not. These results may be due to the numerous factors that can affect lactate quantification, including difficulties with specimen collection, improper specimen handling, and delays in plasma separation and sample processing that may lead to spurious changes in lactate levels (Sacks, 1994). However, the fact that both alanine, which is less sensitive to sampling issues, and lactate are reduced indicates that these results are not artifacts of sample collection.

The technical factors affecting lactate quantification, in addition to the small sample size and potential plasma lactate variations that may be observed with physical activity, are limitations of this study. However, the research subjects were hospitalized during the study and had only minimal physical activities before blood sampling. This in turn can minimize the confounding effect of lactate variability with physical activity. In addition, we have demonstrated that the baseline plasma lactate concentrations before arginine supplementation correlated strongly with the baseline levels before citrulline supplementation, indicating minimal fluctuations in the plasma lactate concentrations. This finding provides more confidence in the measured lactate values and adds more support to the conclusion that the reduced lactate levels are due to the supplementation rather than fluctuations related confounding factors like changes in physical activity and technical sampling factors.

This study adds further evidence to the potential beneficial effect of citrulline and arginine as therapeutic options in MELAS syndrome and other mitochondrial diseases. Further long-term clinical studies are needed to evaluate other aspects of mitochondrial disease that can benefit from restoring NO production with arginine and citrulline supplementation.

In this study we also measured heteroplasmy of the m.3234A>G mutation in both blood and urine samples, and demonstrated positive correlations between lactic acid and heteroplasmy in both blood and urine. Interestingly, the correlation was more significant between the lactate levels and the heteroplasmy in urine. It has been demonstrated in previous studies that the m.3243A>G heteroplasmy percentage is higher in urine than blood and also decreases progressively in blood with aging. Furthermore, in some individuals with MELAS syndrome the m.3243A>G mutation is undetectable in blood but clearly present in urine. Therefore, it has been suggested to use urine sediment cells as the non-invasive cell type of choice to diagnose MELAS syndrome (Shanske et al., 2004, Mehrazin et al., 2009). Our findings of a stronger correlation between heteroplasmy in urine and lactic acidemia support the decision to test heteroplasmy in urine and may indicate a better correlation between heteroplasmy in urine and the clinical severity of MELAS syndrome.

## 5 Conclusions

Citrulline supplementation resulted in improved lactic acidemia in adults with MELAS syndrome. Lower lactate levels were observed after arginine supplementation, but were not statistically significant. These results add more support to the potential therapeutic use of arginine and citrulline in MELAS and suggest a better therapeutic effect with citrulline. Further studies are required to elucidate the effects of long-term supplementation with these compounds.
